# Insurance Status Is Associated with Treatment Allocation and Outcomes after Subarachnoid Hemorrhage

**DOI:** 10.1371/journal.pone.0105124

**Published:** 2014-08-20

**Authors:** Charles Hobson, John Dortch, Tezcan Ozrazgat Baslanti, Daniel R. Layon, Alina Roche, Alison Rioux, Jeffrey S. Harman, Brenda Fahy, Azra Bihorac

**Affiliations:** 1 Department of Surgery, Malcom Randall VA Medical Center, Gainesville, Florida, United States of America; 2 Department of Anesthesiology, University of Florida, Gainesville, Florida, United States of America; 3 Department of Health Services Research, Management and Policy, University of Florida, Gainesville, Florida, United States of America; Johns Hopkins Bloomberg School of Public Health, United States of America

## Abstract

**Objective:**

Subarachnoid hemorrhage (SAH) is a particularly devastating type of stroke which is responsible for one third of all stroke-related years of potential life lost before age 65. Surgical treatment has been shown to decrease both morbidity and mortality after subarachnoid hemorrhage. We hypothesized that payer status other than private insurance is associated with lower allocation to surgical treatment for patients with SAH and worse outcomes.

**Design:**

We examined the association between insurance type and surgical treatment allocation and outcomes for patients with SAH while adjusting for a wide range of patient and hospital factors. We analyzed the Nationwide Inpatient Sample hospital discharge database using survey procedures to produce weighted estimates representative of the United States population.

**Patients:**

We studied 21047 discharges, representing a weighted estimate of 102595 patients age 18 and above with a discharge diagnosis of SAH between 2003 and 2008.

**Measurements:**

Multivariable logistic and generalized linear regression analyses were used to assess for any associations between insurance status and surgery allocation and outcomes.

**Main Results:**

Despite the benefits of surgery 66% of SAH patients did not undergo surgical treatment to prevent rebleeding. Mortality was more than twice as likely for patients with no surgical treatment compared to those who received surgery. Medicare patients were significantly less likely to receive surgical treatment.

**Conclusions:**

Nearly two thirds of patients with SAH don't receive operative care, and Medicare patients were significantly less likely to receive surgical treatment than other patients. Bias against the elderly and those with chronic illness and disability may play a part in these findings. A system of regionalized care for patients presenting with SAH may reduce disparities and improve appropriate allocation to surgical care and deserves prospective study.

## Introduction

Non-traumatic subarachnoid hemorrhage (SAH) is a sudden and devastating hemorrhagic variant of stroke with a high likelihood of death and, if a patient survives, a high likelihood of severe and long term disability. One third of all stroke related years of potential life lost before age 65 are due to SAH, and 46% of people who have suffered SAH have cognitive impairment that affects their subsequent quality of life [1]. The majority (80–85%) of non-traumatic SAH is caused by a ruptured intracerebral aneurysm, and current evidence-based recommendations are for early surgical treatment through either microsurgical clipping or endovascular coiling and ablation of the aneurysm to prevent rebleeding [2]. However there is wide variation in the utilization of these procedures, and consistently only about a third of all SAH patients in the United States actually receive some form of surgical treatment [3]–[7].

Another consistent finding in recent studies is that insurance status has important implications in both allocation to surgery and surgical outcomes. Medicare patients have been shown to be less likely to receive surgery for lung cancer and acute diverticulitis [8], [9]. Insurance status can influence the intensity of utilization of hospital resources after admission for diverse acute medical and surgical diagnoses [10]–[13]. Insurance status has also been demonstrated to influence the decision to transfer patients with severe trauma and traumatic brain injury to tertiary care hospitals [14], [15].

Given the historically low allocation to surgery for patients suffering from SAH, and the recent reports of the association between payer status and both allocation to treatment and outcomes for diverse patient populations, we hypothesized that payer status other than private insurance is associated with lower allocation to surgical treatment for patients with SAH and worse outcomes. We studied that question using a large national administrative database looking for any association between insurance type and surgical treatment allocation and outcomes for patients with SAH, while adjusting for a wide range of patient and hospital factors.

## Methods

### Ethics

The University of Florida institutional review board approved this study (H-323-2008) as an exempt study.

### Data Source

We obtained discharge data for the years 2003–2008 from the Nationwide Inpatient Sample (NIS) database which is maintained by the Agency for Healthcare Research and Quality (AHRQ) as part of the Healthcare Cost and Utilization Project [16]. The NIS database is a 20% stratified random sample of all hospitalization capturing approximately 5–8 million hospital discharges through the Unites States. Since NIS does not contain unique patient identifiers the individual patients cannot be tracked across hospitalizations or time and therefore only discharges and not individual patients can be analyzed. The consistency of coding and variables in the dataset is maintained by AHRQ using extensive preprocessing to reconcile variations in coding by individual states and definitions across them. The NIS is the largest all-payer inpatient care database in the US that includes charge information on all patients, regardless of payer, including patients covered by Medicare, Medicaid, and private insurance, and those who are uninsured on the national level. Data elements include more than 100 clinical variables including patient demographics; primary and secondary diagnoses as captured by International Classification of Diseases, Ninth Revision, Clinical Modification codes (ICD-9-CM); insurance type; discharge disposition; hospital charges and hospital characteristics. The NIS database provides discharge weights to allow researchers to calculate nationally representative estimates [16].

### Inclusion and Exclusion Criteria and Definition of Outcomes

We identified all discharges for adults age 18 and above with a primary or secondary ICD-9-CM diagnosis code 430 for subarachnoid hemorrhage and no concomitant diagnosis of arteriovenous malformation or head trauma, as determined by a previously validated approach utilizing ICD-9-CM diagnostic codes [6] (Methods S1). Patients were stratified by insurance status using NIS variable ‘Primary Payer Group’: Medicare, Medicaid, uninsured and private insurance. The uninsured payer group included the patients who were categorized as “self-pay” and “no charge”. We excluded patients with primary payer status categorized as “other” as this group constitutes a very small yet heterogeneous group of patients with varying insurance coverage (n = 1316, weighted n = 6380, 3.8%) [12]. The data including race information contained a large percentage of missing data (29% missing), while other variables contained much smaller percentages of missing information. Patients with missing race information were considered as a separate group. Missing values were not included in the statistical analyses for other variables. Comorbid conditions on admission were described using the AHRQ comorbidity measures based on the method of Elixhauser et al. using relevant ICD-9-CM diagnostic codes except for end-stage renal disease and chronic kidney disease not requiring renal replacement therapy for which we used updated definitions that can differentiate between these conditions [17]. The Charlson comorbidity index was used as a composite measure for medical comorbidities [18].

All measured outcomes were established prior to data analysis. Primary outcome variables were treatment allocation (type of surgery received or not) and hospital mortality. Surgical treatment of SAH was defined by primary or secondary ICD-9-CM procedure codes for microvascular clipping (39.51 and 39.52), or endovascular coiling (39.72 and 39.79). Secondary outcomes included hospital cost, hospital complications and length of stay. We calculated inflation-adjusted hospital cost by applying hospital cost-to-charge ratios to hospital charges and adjusting for inflation using the Consumer Price Index for Medical Services provided by the US Bureau of Labor Statistics [19]. All hospital complications were limited to events that occurred during the index hospital stay. For the definition of sepsis we used AHRQ patient safety indicators “Postoperative Sepsis” and “Central Venous Catheter-Related Blood Stream Infection” criteria [20]. To identify patients who developed hospital-acquired pneumonia, cardiac arrhythmias and venous thromboembolism we used AHRQ's Clinical Classification Software (CCS) codes 122, 106 and 118, respectively [21]. Mechanical ventilation was defined by ICD-9-CM procedure codes for continuous mechanical ventilation (96.7x). Severe acute kidney injury was defined by the combination of the secondary diagnostic codes for acute renal failure and the procedure codes for renal replacement [22].

### Hospital Characteristics

Hospitals were described based on location (urban vs. rural), teaching status, bed size and regional information included in the NIS database. We characterized hospitals' volume status regarding the annual number of SAH admissions and surgical procedures for SAH using approach developed for administrative databases that defines high-volume SAH hospitals as those with >20 admissions for SAH per year [23]. We used ICD-9-CM procedure codes 00.62 and 39.50 to identify hospitals offering angioplasty for vasospasm as previously validated [24].

### Statistical Analysis

We used SURVEY procedures in SAS (v.9.3, Cary, N.C.) and svy commands in Stata (v 12, StataCorp, College Station, TX) to account for stratification, clustering, and unequal weighting of the NIS survey design. Discharge weights provided in the NIS were used to generate nationally representative estimates for the U.S. population. The Taylor series linearization method was used to estimate standard errors [25]. We followed analytic procedures as recommended by AHRQ for subpopulation analyses [26]. For all statistical analyses, the threshold for significance was 0.05.

Using weighted multivariable logistic and generalized linear regression models we tested for any associations between insurance status and surgical allocation and outcomes, adjusting for patient and hospital factors as explanatory variables. All multivariable analyses were performed using sampling and poststratification weights and hospital clusters to yield nationally representative estimates for the U.S. population with appropriate standard error estimates. For all models explanatory variables were chosen from domains of the Andersen behavioral model of healthcare utilization [27]. Predisposing characteristics included gender, age, and race. Enabling resources included median household income in ZIP code of residence (based on each patient's ZIP Code, with quartiles based on 1999 demographics [16], hospital characteristics and time of admission. Evaluated health need was represented by the Charlson comorbidity index and presence of six major entered comorbid conditions (hypertension, diabetes mellitus, chronic lung disease, congestive heart failure, chronic kidney disease and end-stage renal disease). Multivariable logistic regression model for hospital mortality was additionally adjusted for surgical treatment allocation and hospital complications while regression model for cost also included hospital survival status. Binary outcomes were modeled with multivariable logistic regression models and for each significant covariate we reported odds ratios (OR) with 95% confidence intervals (95% CI). Hospital cost and length of stay were modeled using generalized linear models with gamma and negative binomial-distributed errors, respectively and a log-link function to account for any skewness. Adjusted means for hospital cost and length of stay were reported with 95% CIs for each insurance type. Estimated regression coefficients were exponentiated to represent the cost ratio for each category with respect to the determined reference category for categorical variables or for one unit change in continuous variables. Bonferroni adjustments were used for multiple comparisons. We used the area under the receiver operating characteristics curve (AUC) values and Hosmer-Lemeshow goodness of fit tests to assess model discrimination and fit. Using bootstrapping as a resampling tool we derived 95% CI of AUCs for all logistic regression models.

We performed sensitivity analyses by comparing the effect of a) exclusion of transferred patients to adjust for multiple admissions [6], b) exclusion of Medicare patients age <65 with the highest burden of comorbidities [28], c) inclusion of patients with missing values for race as a separate category, d) exclusion of patients with end-stage renal disease, e) exclusion of patients with SAH as a secondary diagnosis, f) exclusion of patients aged 65 years and older as they are almost exclusively insured covered by Medicare, g) using cut-off of 80 SAH admissions/year to define high volume hospitals and h) omission of different covariates on model fit for each outcome.

## Results

From 2003 to 2008, 21,047 discharges for patients age 18 and above with a diagnosis of SAH were evaluated, representing a weighted estimate of 159,624 patients nationwide (Table 1). Approximately 62% of the cohort was female and the mean age was 59 years old. A majority of the patients had either private insurance (43%) or Medicare (36%). Medicare patients were older, had a higher Charlson comorbidity index compared to other patients and were more likely to have at least one of the major comorbid conditions. Medicare patients were significantly less likely to be admitted to teaching hospitals, to hospitals with high SAH volume and to those offering angioplasty. Ten percent of all SAH patients were transferred to another acute care hospital after initial hospitalization. Among transferred patients, Medicare and Medicaid insurance and larger comorbidity burden were factors significantly associated with less likelihood of transfer to another acute hospital (Table S1).

**Table 1:** Demographics, Socioeconomic Measures, Comorbid Conditions and Treatment Allocation for Patients with Subarachnoid Hemorrhage.

Variables^a^	All insurance	Private	Medicare	Medicaid	Uninsured
Weighted number, n (%)	159,624	68,711 (43)	56,923 (36)	18,090 (11)	15,900 (10)
Age (years), mean (SE)^b^	58.6 (0.2)	51.5 (0.1)	73.8 (0.2)	47.7 (0.3)	47.7 (0.3)
Female, n (%)^b^	99240 (62.3)	41537 (60.7)	37495 (65.9)	11381 (63.1)	8847 (55.8)
Race, n (%)^b^					
White	75584 (47.4)	33190 (48.3)	31263 (54.9)	5465 (30.2)	5666 (35.6)
African-American	16387 (10.3)	6271 (9.1)	4321 (7.6)	3398 (18.7)	2397 (15.1)
Hispanic	14012 (8.8)	4531 (6.6)	3522 (6.2)	3432 (18.9)	2526 (15.9)
Others	9120 (5.7)	3808 (5.5)	2789 (4.9)	1440 (8.0)	1083 (6.8)
Missing	44521 (27.9)	20911 (30.4)	15028 (26.4)	4354 (24.1)	4228(26.6)
Household income at zip code level less than $25,000 annually, n (%)^b^	41631 (26.1)	13346 (19.4)	15314 (26.9)	7376 (40.8)	5595 (35.2)
Weekend admission, n (%)	42498 (26.6)	17977 (26.2)	15159 (26.6)	4986 (27.6)	4377 (27.5)
Comorbid conditions, n (%)					
Hypertension^b^	86189 (54.5)	32282 (47.5)	37120 (65.9)	9062 (50.6)	7726 (48.7)
Diabetes mellitus^b^	21276 (13.3)	6565 (9.6)	10757 (18.9)	2491 (13.8)	1463 (9.2)
Chronic lung disease^b^	17639 (11.2)	5583 (8.2)	8776 (15.6)	2061 (11.5)	1219 (7.7)
Congestive heart failure^b^	9543 (6.0)	2353 (3.5)	5965 (10.6)	848 (4.7)	377 (2.4)
Chronic kidney disease^b^	7240 (4.5)	1630 (2.4)	4572 (8.0)	674 (3.7)	364 (2.3)
End stage renal disease^b^	2878 (1.8)	525 (0.8)	1964 (3.5)	242 (1.3)	147 (0.9)
Charlson comorbidity index, mean (SE)^b^	1.9 (0.01)	1.7 (0.01)	2.2 (0.02)	1.9 (0.03)	1.6 (0.02)
Hospital characteristics, n (%)					
Teaching hospital status^b^	116020 (72.7)	53312 (77.6)	36622 (64.3)	14398 (79.7)	11688 (73.5)
High SAH volume^b^	113794 (71.3)	53033 (77.2)	34578 (60.7)	13921 (77.0)	12262 (77.1)
Hospital offering angioplasty^b^	91860 (57.5)	43837 (63.8)	27437 (48.2)	11199 (61.9)	9387 (59.0)
Treatment Group, n (%)^b^					
No Surgical Treatment	105471 (66.1)	41288 (60.1)	44319 (77.9)	10315 (57.0)	9548 (60.1)
Microsurgical Clipping	31068 (19.5)	16064 (23.4)	6369 (11.2)	4736 (26.2)	3899 (24.5)
Endovascular Coiling	23085 (14.5)	11358 (16.5)	6236 (11.0)	3038 (17.0)	2452 (15.4)
Transferred on discharge to another acute care hospital, n (%))^b^	16253 (10.2)	7603 (11.1)	5104 (9.0)	1762 (9.7)	1784 (11.2)

Abbreviations: SE, standard error.

a All values were estimated to yield nationally representative estimates for the U.S. population (see Methods).

b P<0.001 when comparing difference between groups using the Wald chi-square test for categorical variables and analysis of variance for continuous variables.

### Allocation to Surgery

Almost two thirds of patients (66.1%) received no surgical treatment to prevent rebleeding, while 19.5% had craniotomy and microsurgical clipping of an aneurysm and 14.5% had endovascular treatment of an aneurysm. Even after adjustment for explanatory variables, and compared to patients with private insurance, Medicare patients were almost 45% less likely to undergo surgical treatment (OR 0.69, CI 0.64–0.75, Table 2). This effect was independent of age and comorbidity burden and remained significant in multiple sensitivity analyses (Table S2). Patients with any degree of chronic kidney disease including end-stage renal disease or with diabetes mellitus were up to two times less likely to undergo surgical treatment compared to patients without. Every year of increasing age was significantly associated with lower allocation to surgery (OR 0.99, CI 0.98–0.99). Patients with other comorbid conditions, on the other hand, including congestive heart failure, hypertension or chronic lung disease were more likely to undergo surgical treatment compared to patients without (OR 1.16–1.47) – these associations remained significant in sensitivity analyses. Patients treated at teaching hospitals, hospitals that see a high volume of SAH patients, and hospitals that offer angioplasty were all more likely to undergo surgery (OR 1.38–3.00).

**Table 2:** Adjusted Odds of Allocation to Surgical Treatment for Subarachnoid Hemorrhage.

Variables	Allocation to surgical treatment Adjusted Odds Ratio (95% confidence interval)^a^	
	All Ages ≥18 years	All Ages ≥18 years after excluding transfers
Weighted number	159,624	143,371
Insurance (Reference Private)		
Medicare	0.69 (0.64, 0.75)	0.68 (0.63, 0.75)
Medicaid	1.09 (0.99, 1.19)	1.07 (0.98, 1.16)
Uninsured	1.01 (0.91, 1.13)	1.01 (0.90, 1.12)
Age (per year)	0.99 (0.98, 0.99)	0.98 (0.98, 0.99)
Female (Reference Male)	1.71 (1.62, 1.80)	1.76 (1.66, 1.86)
Comorbid conditions (Reference none)		
End stage renal disease	0.50 (0.39, 0.63)	0.52 (0.41, 0.67)
Chronic kidney disease	0.63 (0.53, 0.76)	0.65 (0.50, 0.71)
Diabetes mellitus	0.64 (0.59, 0.71)	0.64 (0.58, 0.70)
Congestive heart failure	1.16 (1.02, 1.33)	1.15 (1.01, 1.31)
Hypertension	1.21 (1.14, 1.29)	1.23 (1.16, 1.31)
Chronic lung disease	1.47 (1.36, 1.59)	1.48 (1.37, 1.61)
Hospital characteristics		
Teaching hospital (reference non-teaching)	1.38 (1.15, 1.65)	1.28 (1.08, 1.52)
High SAH volume (Reference low volume)	3.00 (2.49, 3.62)	2.90 (2.43, 3.47)
Hospital offering angioplasty (Reference not offering)	2.10 (1.78, 2.49)	1.83 (1.57, 2.13)

Table shows only variables with at least 0.05 significance level in one of the models.

a Odds ratios were calculated from weighted multivariable logistic regression, yielding nationally representative estimates for the U.S. population The models included demographic and socioeconomic information, hospital characteristics, and comorbid conditions described in the methods. Areas under the curves (95% confidence interval) were 0.76 (0.73, 0.79) and 0.75 (0.71, 0.78) for the two models, respectively.

### Hospital Complications and Mortality

Almost half of all patients had at least one major hospital complication. The most common complications were mechanical ventilation, cardiac arrhythmias and hospital-acquired pneumonia (Table S3). Patients with Medicare and Medicaid insurance had increased adjusted odds for most of the complications, even after adjusting for age, comorbidity burden and surgical treatment (Table S4). Unadjusted hospital mortality was 24%, with the highest mortality seen in patients with Medicare (32%) and the lowest in patients with private insurance (18%). Medicare, Medicaid, and uninsured patients all had higher adjusted mortality when compared to patients with private insurance (OR 1.12–1.82) (Table 3). This association was independent of age, comorbidity burden, surgical treatment and hospital complications and remained significant in multiple sensitivity analyses even after exclusion of transfers, exclusion of patients older than 64, exclusion of Medicare patients or after application of different cut-off for high SAH volume hospitals. When excluding patients transferred from one institution to another the mortality difference between Medicare and privately insured patients was no longer statistically significant. Patients who did not undergo surgical treatment had more than twice the adjusted mortality of those who did have surgery (OR 2.59, 95% CI 2.37–2.82). Chronic kidney disease was the only comorbid condition independently associated with an increase in hospital mortality, and that effect remained consistent in all sensitivity analyses (Table 3 and Table S5).

**Table 3:** Adjusted Odds of Hospital Mortality after Subarachnoid Hemorrhage.

Variables	Hospital mortality Adjusted Odds Ratio (95% confidence interval)^a^	
	All Ages ≥18 years	All Ages ≥18 years after excluding transfers
Weighted number	159,624	143,371
Insurance (Reference Private)		
Medicare	1.12 (1.01, 1.23)	1.08 (0.97, 1.19)
Medicaid	1.23 (1.10, 1.38)	1.19 (1.06, 1.33)
Uninsured	1.82 (1.62, 2.04)	1.81 (1.61, 2.04)
Age (per year)	1.02 (1.01, 1.02)	1.02 (1.01, 1.02)
Female (Reference Male)	1.22 (1.15, 1.30)	1.24 (1.17, 1.32)
Comorbid conditions (Reference none)		
Chronic kidney disease	1.24 (1.06, 1.45)	1.20 (1.02, 1.43)
Chronic lung disease	0.88 (0.79, 0.98)	0.90 (0.81, 0.99)
Congestive heart failure	0.79 (0.70, 0.89)	0.78 (0.68, 0.88)
Hospital characteristics		
Teaching hospital (reference non-teaching)	0.92 (0.85, 1.00)	0.88 (0.80, 0.96)
High SAH volume (Reference low volume)	0.99 (0.9, 1.10)	0.88 (0.79, 0.97)
Hospital offering angioplasty (Reference not offering)	0.90 (0.82, 1.00)	0.80 (0.73, 0.89)
No surgical treatment (Reference surgical treatment)	2.59 (2.37, 2.82)	2.91 (2.67, 3.17)
Hospital complications (Reference none)		
One	7.40 (6.84, 8.02)	7.82 (7.20, 8.48)
Two	8.38 (7.57, 9.29)	8.63 (7.77, 9.59)
Greater or equal to three	7.54 (6.55, 8.68)	7.50 (6.52, 8.64)

Table shows only variables with at least 0.05 significance level in one of the models.

a Odds ratios were calculated from weighted multivariable logistic regression, yielding nationally representative estimates for the U.S. population. The models included demographic and socioeconomic information, hospital characteristics, and comorbid conditions described in the methods. Areas under the curves (95% confidence interval) were 0.81 (0.77, 0.83) and 0.82 (0.79, 0.85) for the two models, respectively.

### Hospital Length of Stay and Costs

The adjusted hospital length of stay for the overall cohort was 12 days (95% CI 11–12). Medicaid patients had three days longer adjusted hospital stay even after adjustment for all other covariates including survival status. Only uninsured patients had significantly less odds of being discharged to nursing home or rehabilitation facilities compared to all other insurance groups (Table S4). The adjusted hospital cost for the overall cohort was $46,953 (95% CI $44,951–48,956). Patients with Medicaid had 11% higher adjusted hospital costs compared to patients with private insurance, while patients with Medicare and the uninsured had 9–13% lower costs (Table 4). Increasing age of the patient and low household income was associated with lower adjusted cost while higher comorbidity index and occurrence of any hospital complication had significantly higher adjusted cost. Patients who underwent surgery incurred over twice the costs of those who did not have surgery while those who died in the hospital had much lower costs. These findings were consistent even after excluding patients who were transferred at hospital discharge.

**Table 4:** Adjusted hospital cost ratios after subarachnoid hemorrhage.

Variables	Adjusted Cost Ratio (95% confidence interval)^a^	
	All Ages ≥18 years	All Ages ≥18 years after excluding transfers
Weighted N	159,624	143,371
Insurance (Reference Private)		
Medicare	0.91 (0.88, 0.95)	0.91 (0.88, 0.95)
Medicaid	1.11 (1.06, 1.16)	1.10 (1.05, 1.16)
Uninsured	0.83 (0.79, 0.88)	0.84 (0.79, 0.88)
Age (per year increase)	0.995 (0.994, 0.996)	0.995 (0.994, 0.996)
Household income at zip code level less than $25,000 annually (Reference >$25,000)	0.9 (0.86, 0.94)	0.9 (0.86, 0.94)
Comorbid conditions (Reference none)		
Diabetes Mellitus	0.96 (0.92, 0.99)	0.95 (0.91, 0.98)
Congestive heart failure	1.06 (1.00, 1.12)	1.06 (1.002, 1.13)
Charlson Comorbidity Index (per unit increase)	1.06 (1.05, 1.07)	1.05 (1.04, 1.07)
Hospital characteristics		
Teaching hospital (Reference non-teaching)	1.16 (1.07, 1.25)	1.14 (1.06, 1.23)
High SAH volume (Reference low volume)	1.35 (1.23, 1.49)	1.30 (1.18, 1.42)
Hospital offering angioplasty (Reference not offering)	1.29 (1.16, 1.44)	1.25 (1.12, 1.39)
Surgery (vs. No surgery)	2.41 (2.30, 2.52)	2.29 (2.19, 2.40)
Hospital complications (per each complication)	1.73 (1.69, 1.77)	1.72 (1.68, 1.76)
Death in hospital (Reference no)	0.57 (0.55, 0.59)	0.54 (0.51, 0.56)

Table shows only variables with at least 0.05 significance level in one of the models.

a Adjusted cost ratio gives the ratio of the expected cost of a group with respect to the reference category. An adjusted cost ratio of 1.5 means that the expected cost is 150% of the reference category. Adjusted cost ratios were calculated using coefficients that were obtained with weighted multivariable gamma regression, yielding nationally representative estimates for the U.S. population. The models included demographic and socioeconomic information, hospital characteristics, comorbid conditions and hospital mortality status as described in the methods.

## Discussion

In a representative national sample of patients age 18 and older diagnosed with SAH between 2003 and 2008 a 66% majority did not undergo surgical treatment to prevent rebleeding, a percentage similar to previous reports in the literature despite ongoing improvements in the care of the patient with SAH, and resulting in mortality more than twice that seen in patients who received surgery [1], [6]. In both unadjusted results and after adjustment for explanatory variables, and in all sensitivity analyses, the Medicare patient population was significantly less likely to receive surgical treatment compared to the other payer groups independent of age and comorbidity status. The Medicare population is heterogeneous and composed of people over the age of 65 and/or with chronic illness or disability.

The finding that Medicare patients were less likely to receive surgery for SAH may be related to our finding that patients with certain chronic illnesses were much less likely to receive surgery. In multiple sensitivity analyses the low odds of receiving operative treatment were consistent for patients with chronic kidney disease of any severity, end-stage renal disease or diabetes mellitus. Patients with chronic kidney disease and end-stage renal disease have been shown to be much less likely to receive appropriate medical management after cardiovascular events [29]. Patients with end-stage renal disease have delayed access to renal transplantation based on their insurance status, with the racial/ethnic minority Medicare patient having 10% the chance of pre-dialysis transplant listing compared to the white privately insured patient – with insurance status the most important factor in that disparity [30]. Interestingly, patients with congestive heart failure, hypertension and chronic lung disease all were more likely to be allocated to surgical treatment, perhaps due to the different perception providers have about these conditions and their prognosis [31].

Medicare beneficiaries under the age 65, 16% of the total Medicare population, have coverage due to either a chronic disability or chronic illness. Patients with disability Medicare entitlement are less likely to receive surgery for early non-small cell lung cancer compared to those without disability, and have a higher cancer-specific mortality [32]. Women with Social Security Disability Insurance and Medicare between the ages of 21 and 64 with breast cancer were less likely to undergo breast conserving surgery, less likely to receive appropriate radiation therapy after surgery, and had lower all-cause survival compared to those without disability[33]. People with disabilities are consistently noted to have worse health than the general population, to experience more chronic conditions unrelated to the disability, and to have lower rates of preventative health services such as blood pressure and cholesterol checks and cancer screening [34].

The finding that Medicare patients were less likely to receive surgery for SAH was independent of patient age, however increasing age was independently associated with lower allocation to surgical treatment. Older patients have less access to curative surgery for gynecologic surgery [35], to appropriate adjuvant therapy for colon cancer [36] and to appropriate rehabilitation therapies after myocardial infarction [37]. Elderly patients with head trauma are noted to take longer to get CT head imaging and are less likely to be transferred for neurosurgical care compared to younger patients [38]. Age bias has been noted to be a factor in lower rates and lower quality of rehabilitative services offered to stroke victims [39].

Health disparities in people with chronic disease, disability or advanced age are thought to be due to individual factors such as biology and patient (and family) preference, to systemic factors such as social and geographic discrimination, and to healthcare factors such as provider behavior and medical facility inaccessibility [40], [41]. It is very unlikely that payer status itself influenced physician decision-making, owing to the urgent nature of treating SAH. More likely is that Medicare coverage is a proxy for bias, either conscious or unconscious, against the elderly and those with chronic illness or disability [42]. Even for patients with the ability to pay there can be provider-based prejudices that prevent a patient from receiving appropriate care [31], [43]. Poor understanding by the patient, or the patient's family if the patient is incapacitated, of the importance of an intervention can contribute to inadequate care [44]. One way to address this kind is to establish norms and guidelines for treatment of relatively rare but devastating conditions such as SAH. Guidelines by both the Neurocritical Care Society and the American Heart Association recommend that patients with SAH be treated at high-volume centers, and that low volume hospitals should consider early transfer of these patients [2], [45]. Our findings substantiate the growing body of literature supporting rapid referral for patients with SAH to centers offering comprehensive treatment modalities such as endovascular therapy, neurocritical intensive care units and specialized rehabilitation services [23], [46], [47]. A system of regionalized care for patients presenting with SAH may improve overall allocation to surgical care while reducing disparity associated with payer status and deserves prospective study.

This study has some limitations. The retrospective nature of this study design makes selection bias one potential concern. The large and representative sampling of our database likely minimizes the effect of selection bias. It is possible that, despite our attempts to adjust for all possible confounders, we may not have measured some variables that contribute to the size of the associations that we demonstrate. Though we controlled for comorbidity in our adjusted models, it is possible that one or more unmeasured comorbidities acting as confounding factors may have been omitted from our analysis. It is also possible that some patients with more advanced comorbid disease were judged not to be operative candidates for surgical treatment of their SAH. Furthermore there is a lack of clinical information on both the severity of SAH and on neurological outcomes after SAH. We are thus not able to determine how our results may have varied with differing levels of clinical SAH severity, and we are limited to assessing only hospital adverse outcomes and costs. The NIS database does not allow us to tell whether patients with a discharge of SAH actually have an aneurysm. However only an estimated 15% of patients with SAH have hemorrhage due to causes other than ruptured aneurysms, those causes including arteriovenous malformation, hypertensive hemorrhage, intracranial neoplasms, and coagulopathy [6]. As we cannot account for patients who suffered devastating strokes and were thus offered only comfort care, and then died soon after transfer to hospice or skilled nursing facility, our results may underestimate the mortality after SAH. The discharge data in the NIS database does not distinguish between patients with a unique hospitalization and patients who have undergone recurrent hospitalizations for the same event. We examined this liability with multiple sensitivity analysis using transfer to another hospital, and showed that our results were not affected. The NIS dataset misses some potentially important explanatory variables from the Andersen behavioral model of utilization, including marital status, education, literacy, employment, family size, religion, and cultural beliefs. To maximize the internal validity of our study we used survey methodology for multivariable analyses and multiple sensitivity analyses addressing missing values, selection bias and the effect of multiple inclusions by elimination of transfer patients.

In conclusion, despite ongoing improvements in the care of the patient with SAH, nearly 2/3 of patients don't receive operative care and Medicare patients in particular were significantly less likely to receive surgical treatment. Bias, both conscious and unconscious, against the elderly and those with chronic illness and disability may play a part in these findings. A system of regionalized care for patients presenting with SAH may reduce disparities and improve appropriate allocation to surgical care and deserves prospective study.

## Supporting Information

Table S1 **Adjusted odds of transfer from the initial admitting hospital to another acute care hospital for insurance status and admission comorbidities for patients with subarachnoid hemorrhage.**
(DOCX)

Table S2 **Association between allocation to surgical treatment and insurance status and comorbidities for patients with subarachnoid hemorrhage: sensitivity analyses.**
(DOCX)

Table S3 **Association between mortality and insurance status and comorbidities after subarachnoid hemorrhage: sensitivity analyses.**
(DOCX)

Table S4 **Adjusted outcomes after subarachnoid hemorrhage for insurance status.**
(DOCX)

Table S5 **Association between mortality and insurance status and comorbidities after subarachnoid hemorrhage: sensitivity analyses.**
(DOCX)

Methods S1 **Detailed description of methods.**
(DOCX)
