# Supplementary material for: Insurance Status Is Associated with Treatment Allocation and Outcomes after Subarachnoid Hemorrhage
Source: PLoS One. 2014 Aug 20;9(8):e105124. doi: 10.1371/journal.pone.0105124 (PMC4139299; doi:10.1371/journal.pone.0105124)
Supplement: Table S1 — Adjusted odds of transfer from the initial admitting hospital to another acute care hospital for insurance status and admission comorbidities for patients with subarachnoid hemorrhage. (DOCX) [file pone.0105124.s001.docx]

Table S1. Adjusted odds of transfer from the initial admitting hospital to another acute care hospital for insurance status and admission comorbidities for patients with subarachnoid hemorrhage

|  | Transfer From the Initial Admitting Hospital to Another Acute Care Hospital  Adjusted Odds Ratio (95% Confidence Interval)^a^ |
| --- | --- |
| Variables | All Ages ≥ 18 |
| Weighted number | 159,624 |
| Insurance |  |
| Medicare | 0.80 (0.71, 0.90) |
| Medicaid | 0.81 (0.69, 0.96) |
| Uninsured | 0.90 (0.77, 1.05) |
| Private | 1.00 (Reference) |
| Age | 0.99 (0.98, 0.99) |
| Charlson Comorbidity Index (per unit increase) | 0.91 (0.87, 0.95) |
| Hospital characteristics |  |
| Non-teaching hospital status (reference. teaching) | 1.32 (1.13, 1.54) |
| Low SAH volume (reference high volume ) | 1.83 (1.51, 2.22) |
| Hospital not offering angioplasty (reference offering) | 2.80 (2.06, 3.78) |
| Area under the curve (95% confidence interval) | 0.72 (0.66, 0.73) |

High-volume subarachnoid hemorrhage (SAH) hospitals are those with >20 admissions for SAH per year.

^a^ Odds ratios were calculated using weighted multivariable logistic regression to yield nationally representative estimates for the U.S. population. The models included demographic and socioeconomic information, hospital characteristics, and comorbid conditions described in the text.
