# Supplementary material for: Insurance Status Is Associated with Treatment Allocation and Outcomes after Subarachnoid Hemorrhage
Source: PLoS One. 2014 Aug 20;9(8):e105124. doi: 10.1371/journal.pone.0105124 (PMC4139299; doi:10.1371/journal.pone.0105124)
Supplement: Table S2 — Association between allocation to surgical treatment and insurance status and comorbidities for patients with subarachnoid hemorrhage: sensitivity analyses. (DOCX) [file pone.0105124.s002.docx]

Table S2. Association between allocation to surgical treatment and insurance status and comorbidities for patients with subarachnoid hemorrhage: sensitivity analyses

| Variables | Allocation to surgical treatment  Adjusted Odds Ratio (95% Confidence Interval) ^a^ | | | | | | | | |
| --- | --- | --- | --- | --- | --- | --- | --- | --- | --- |
|  | All Ages ≥ 18 | All Ages ≥ 18 excluding transfers | Ages ≥ 18 excluding Medicare | Ages ≥ 18 excluding Medicare and transfers | Ages ≥ 18 using cut-off > 80 SAH/year for high volume hospitals | Age 18-64 | Age 18-64  excluding transfers | Age 18-64  Excluding Medicare | Age 18-64  Excluding Medicare and transfers |
| Weighted N | 159,624 | 143,371 | 102,700 | 91,551 | 159,624 | 102,595 | 91,413 | 94,886 | 84,577 |
| Insurance |  |  |  |  |  |  |  |  |  |
| Medicare | 0.69 (0.63, 0.75) | 0.68 (0.63, 0.75) | excluded | excluded | 0.68 (0.63, 0.74) | 0.67 (0.59, 0.76) | 0.66 (0.58, 0.75) | excluded | excluded |
| Medicaid | 1.09 (0.99, 1.19) | 1.07 (0.98, 1.16) | 1.11 (1.01, 1.21 ) | 1.09 (0.99, 1.19) | 1.08 (0.99, 1.18) | 1.15 (1.06, 1.25) | 1.13 (1.04, 1.23) | 1.13 (1.04, 1.24) | 1.12 (1.02, 1.22) |
| Uninsured | 1.01 (0.91, 1.13) | 1.01 (0.90, 1.12) | 1.04 (0.93, 1.16) | 1.03 (0.92, 1.15) | 1.04 (0.93, 1.15) | 1.05 (0.94, 1.16) | 1.04 (0.94, 1.16) | 1.03 (0.92, 1.15) | 1.02 (0.92, 1.14) |
| Private | 1.00 (Reference) | 1.00 (Reference) | 1.00 (Reference) | 1.00 (Reference) | 1.00 (Reference) | 1.00 (Reference) | 1.00 (Reference) | 1.00 (Reference) | 1.00 (Reference) |
| Comorbid Conditions (reference none) |  |  |  |  |  |  |  |  |  |
| End stage renal disease | 0.50 (0.39, 0.63) | 0.52 (0.41, 0.67) | 0.52 (0.36, 0.75) | 0.54 (0.37, 0.80) | 0.48 (0.38, 0.61) | 0.52 (0.36, 0.74) | 0.54 (0.37, 0.79) | 0.54 (0.36, 0.80) | 0.57 (0.37, 0.86) |
| Chronic kidney disease | 0.63 (0.53, 0.76) | 0.65 (0.50, 0.71) | 0.63 (0.50, 0.81) | 0.59 (0.46, 0.76) | 0.62 (0.52, 0.74) | 0.64 (0.52, 0.81) | 0.59 (0.47, 0.74) | 0.64 (0.51, 0.83) | 0.59 (0.46, 0.76) |
| Diabetes mellitus | 0.64 (0.59, 0.71) | 0.64 (0.58, 0.70) | 0.61 (0.54, 0.68) | 0.59 (0.53 0.66) | 0.65 (0.59, 0.71) | 0.59 (0.53, 0.66) | 0.57 (0.51, 0.64) | 0.58 (0.52, 0.65) | 0.57 (0.5, 0.64) |
| Congestive heart failure | 1.16 (1.02, 1.33) | 1.15 (1.01, 1.31) | 1.26 (1.06,1.50) | 1.24 (1.03, 1.50) | 1.16 (1.02, 1.33) | 1.29 (1.08, 1.53) | 1.27 (1.06, 1.52) | 1.38 (1.14, 1.66) | 1.35 (1.11, 1.64) |
| Hypertension | 1.21 (1.14, 1.29) | 1.23 (1.16, 1.31) | 1.30 (1.21, 1.39) | 1.33 (1.24, 1.42) | 1.23 (1.16, 1.31) | 1.29 (1.21, 1.38) | 1.32 (1.24, 1.41) | 1.30 (1.21, 1.40) | 1.32 (1.23, 1.42) |
| Chronic lung disease | 1.47 (1.36, 1.59) | 1.48 (1.37, 1.61) | 1.38 (1.27, 1.54) | 1.38 (1.24, 1.55) | 1.49 (1.37, 1.61) | 1.36 (1.23, 1.52) | 1.37 (1.23, 1.53) | 1.33 (1.19, 1.49) | 1.34 (1.19, 1.50) |

^a^ Odds ratios were calculated using weighted multivariable logistic regression to yield nationally representative estimates for the U.S. population.

The models included demographic and socioeconomic information, hospital characteristics, and comorbid conditions described in the text.
