# Supplementary material for: Insurance Status Is Associated with Treatment Allocation and Outcomes after Subarachnoid Hemorrhage
Source: PLoS One. 2014 Aug 20;9(8):e105124. doi: 10.1371/journal.pone.0105124 (PMC4139299; doi:10.1371/journal.pone.0105124)
Supplement: Table S3 — Association between mortality and insurance status and comorbidities after subarachnoid hemorrhage: sensitivity analyses. (DOCX) [file pone.0105124.s003.docx]

Table S3. Unadjusted hospital outcomes after subarachnoid hemorrhage.

| Variables | All insurance | Private | Medicare | Medicaid | Uninsured |
| --- | --- | --- | --- | --- | --- |
| Weighted N | 159,624 | 68,711 | 56,923 | 18,090 | 15,900 |
| Mechanical ventilation, % (SE)^a^ | 33.3 (0.6) | 29.9 (0.7) | 34.6 (0.7) | 39.1 (1) | 36.4 (1.4) |
| Cardiac arrhythmias, % (SE)^a^ | 14.9 (0.3) | 10.7 (0.4) | 22.8 (0.4) | 10.2 (0.5) | 10.5 (0.6) |
| Hospital-acquired pneumonia, % (SE)^a^ | 10.0 (0.3) | 9.1 (0.3) | 11.0 (0.5) | 12.5 (0.6) | 7.8 (0.7) |
| Severe acute kidney injury, % (SE)^a^ | 3.9 (0.1) | 2.7 (0.2) | 5.3 (0.2) | 4.9 (0.4) | 3.3 (0.4) |
| Severe sepsis, % (SE)^a^ | 3.9 (0.2) | 3.3 (0.2) | 4.1 (0.2) | 6.0 (0.4) | 3.3 (0.4) |
| Venous thromboembolism, % (SE)^a^ | 2.2 (0.1) | 2.5 (0.2) | 1.9 (0.2) | 2.3 (0.3) | 1.6 (0.3) |
| Number of complications, % (SE)^a^ |  |  |  |  |  |
| None | 52 (0.5) | 59 (0.6) | 45 (0.6) | 50 (1.0) | 55 (1.0) |
| One | 32 (0.3) | 28 (0.4) | 37 (0.5) | 31 (0.8) | 32 (1.0) |
| Two | 12 (0.2) | 10 (0.3) | 14 (0.3) | 14 (0.6) | 11 (0.6) |
| Three and greater | 4 (0.1) | 3 (0.2) | 5 (0.2) | 5 (0.4) | 3 (0.2) |
| Hospital mortality, % (SE)^a^ | 24 (0.4) | 18 (0.4) | 32 (0.6) | 21 (0.8) | 26 (1.1) |
| Discharge to nursing home, % (SE)^a^ | 26 (0.5) | 22 (0.6) | 34 (0.7) | 24 (0.9) | 13 (1.0) |
| Length of stay (days), Mean (SE)^a^  Median (25th, 75th)  Range | 12 (0.2)  8 (2, 16)  0-326 | 12 (0.2)  9 (3, 16)  0-326 | 10 (0.2)  6 (2, 14)  0-137 | 17 (0.5)  11 (3, 20)  0-268 | 11 (0.4)  8 (2, 15)  0-175 |
| Hospital cost (1000 $), Mean (SE)^a^  Median (25th, 75th)  Range | 45 (2)  26 (9, 61)  0.03-545 | 50 (2)  33 (13, 67)  0.03-545 | 36 (2)  16 (6-48)  0.03-496 | 62 (3)  42 (15-84)  0.5-542 | 40 (2)  26 (10, 54)  0.4-523 |

Abbreviations: SE: standard error.

^a^P-value<0.001 for comparison among primary payer groups using Wald chi-square test for categorical and analysis of variance for continuous variables.
