# Supplementary material for: Insurance Status Is Associated with Treatment Allocation and Outcomes after Subarachnoid Hemorrhage
Source: PLoS One. 2014 Aug 20;9(8):e105124. doi: 10.1371/journal.pone.0105124 (PMC4139299; doi:10.1371/journal.pone.0105124)
Supplement: Table S4 — Adjusted outcomes after subarachnoid hemorrhage for insurance status. (DOCX) [file pone.0105124.s004.docx]

Table S4. Adjusted outcomes after subarachnoid hemorrhage for insurance status

| Adjusted outcomes^a^ | Medicare  (Weighted N=56,923) | Medicaid  (Weighted N=18,090) | Uninsured  (Weighted N=15,900) | Private  (Weighted N=68,711) |
| --- | --- | --- | --- | --- |
| Mechanical ventilation  (Adjusted OR, 95% CI) | 1.12 (1.04, 1.20) ^c^ | 1.42 (1.30, 1.54)^b^ | 1.32 (1.20, 1.46)^b^ | 1.00 (Reference) |
| Cardiac arrhythmias  (Adjusted OR, 95% CI) | 1.22 (1.11, 1.34)^b^ | 1.05 (0.93, 1.18) | 1.11 (0.97, 1.28) | 1.00 (Reference) |
| Hospital-acquired pneumonia  Adjusted (OR, 95% CI) | 1.05 (0.93, 1.19) | 1.37 (1.22, 1.55)^b^ | 0.86 (0.73, 1.02) | 1.00 (Reference) |
| Severe acute kidney injury (Adjusted OR, 95% CI) | 1.23 (1.02, 1.49)^d^ | 1.61 (1.32, 1.97)^b^ | 1.22 (0.94, 1.58) | 1.00 (Reference) |
| Severe sepsis  (Adjusted OR, 95% CI) | 1.12 (0.96, 1.31) | 1.67 (1.40, 2.00)^b^ | 0.94 (0.75, 1.18) | 1.00 (Reference) |
| Venous thromboembolism  (Adjusted OR, 95% CI) | 0.86 (0.69, 1.08) | 0.98 (0.74, 1.30) | 0.70 (0.51, 0.95)^d^ | 1.00 (Reference) |
| Discharge to nursing home ( Adjusted OR, 95% CI) | 1.39 (1.27, 1.52)^b^ | 1.31 (1.18, 1.46)^b^ | 0.67 (0.56, 0.80)^b^ | 1.00 (Reference) |
| Length of Stay (days)  Adjusted mean (95% CI) | 11.3 (11.0, 11.6) | 14.6 (14.4, 15.0)^b^ | 11.2 (10.9, 11.5) | 11.6 (11.2, 11.9) |
| Hospital cost (1000 $)  Adjusted mean (95% CI) | 44 (42, 46)^b^ | 54 (51, 56)^b^ | 40 (38, 42)^b^ | 48 (45, 49) |

Abbreviations: CI, confidence interval; OR, odds ratio

^a^Odds ratios and adjusted means for cost and length of stay were calculated using weighted multivariable logistic regression, gamma regressions and negative binomial regressions, respectively, to yield nationally representative estimates for the U.S. population. The models included demographic and socioeconomic information, hospital characteristics, comorbid conditions and hospital mortality status as described in the methods.

Area under the curve ranged between 0.62 and 0.81 for logistic regression models.

^b^P-value<0.001, ^c^P-value<0.01 and ^d^P-value<0.05 comparing to private insurance group.
