# Supplementary material for: Insurance Status Is Associated with Treatment Allocation and Outcomes after Subarachnoid Hemorrhage
Source: PLoS One. 2014 Aug 20;9(8):e105124. doi: 10.1371/journal.pone.0105124 (PMC4139299; doi:10.1371/journal.pone.0105124)
Supplement: Methods S1 — Detailed description of methods. (DOCX) [file pone.0105124.s006.docx]

**Methods S1. Detailed description of methods.**

**Data Source**

We obtained data for the years 2003-2008 from the Nationwide Inpatient Sample (NIS) hospital discharge database which is maintained by Agency for Healthcare Research and Quality (Rockville, MD) as part of the Healthcare Cost and Utilization Project (HCUP) [1]. The NIS database is a 20% stratified random sample of all hospital inpatient discharges from approximately 1000 hospitals from 40 states, capturing approximately 5–8 million hospital discharges through the Unites States. Geographic region, urban or rural location, teaching status, ownership, and bed size of hospitals were used as stratification variables. It contains in-depth information on patient demographics, payment source, total charges, length of stay, hospital characteristics, and outcomes. Since NIS does not contain unique patient identifiers the individual patients cannot be tracked across hospitalizations or time and therefore only discharges and not individual patients can be analyzed. The consistency of coding and variables in the dataset is maintained by AHRQ using extensive preprocessing to reconcile variations in coding by individual states and definitions across them. Data contain elements summarized from the hospital discharge abstract of universal billing form 92 and are similar to other commonly used administrative data sources, such as Medicare Part A.

The NIS is the largest all-payer inpatient care database in the US that includes charge information on all patients, regardless of payer, including patients covered by Medicare, Medicaid, and private insurance, and those who are uninsured on the national level. Among more than 100 clinical variables each index admission includes up to 15 diagnosis and procedure International Classification of Diseases, 9th edition, Clinical Modification (ICD-9-CM) codes. The NIS database provides discharge weights which can be used to derive US national estimates from the representative sample of patients [1-3]. An overview of the NIS is available at <http://www.hcup-us.ahrq.gov/nisoverview.jsp>.

**Inclusion and Exclusion Criteria**

A weighted total number of 159,624 discharges with a primary or secondary diagnosis of subarachnoid hemorrhage were identified by the ICD-9-CM diagnosis code 430 for subarachnoid hemorrhage. Patients with a primary or secondary diagnosis of arteriovenous malformation (ICD-9-CM code 747.81) or head trauma (ICD-9-CM codes 801.00-803.20, 850.5-853.06, and 873.0-873.44) were excluded. [4]

**Patient Characteristics**

Patients were stratified by insurance status using NIS variable ‘Primary Payer Group’: Medicare, Medicaid, uninsured and private insurance. The uninsured payer group included the patients who were categorized as “self-pay” and “no charge”. We excluded patients with primary payer status categorized as “other” as this group constitutes a very small yet heterogeneous group of patients with varying insurance coverage (n=1316, weighted n=6380, 3.8%) [2]. The data including race information contained a large percentage of missing data (29% missing), while other variables contained much smaller percentages of missing information. Patients with missing race information were considered as a separate group. Missing values were not included in the statistical analyses for other variables. Comorbid conditions on admission were described using the AHRQ comorbidity measures included in the NIS data elements and are based on the method of Elixhauser et al. using relevant ICD-9-CM diagnostic codes except for end-stage renal disease (ESRD) and chronic kidney disease (CKD) not requiring renal replacement therapy (RRT) for which we used updated definitions that can differentiated between these conditions [5]. ICD-9-CM diagnostic codes for comorbidities are available at <http://www.hcup-us.ahrq.gov/toolssoftware/comorbidity/Table2-FY2012-V3_7.pdf>. ESRD on admission was identified by the ICD9-CM diagnostic codes (V45.1, V56.0, V56.8) or by combination of RRT procedure codes (54.98 and 39.95) with CKD diagnostic codes (403.x, 404.x, 581.x-586.x and 593.9) but no concomitant code for acute renal failure [6]. Since many other comorbidities had low prevalence in the study population (<2%), including cancer and dementia, we used Charlson comorbidity index as a composite measure for medical comorbidities [7] included in all multivariable analyses.

**Definition of Outcomes**

All measured outcomes were established prior to data analysis. Information on hospital mortality, length of stay, and hospital charges are included in the NIS database. Primary outcomes were treatment allocation (type of surgery received or not) and hospital mortality. Surgical treatment of SAH was defined by primary or secondary ICD-9-CM procedure codes for microvascular neurosurgical clipping (ICD-9-CM codes 39.51 (clipping of aneurysm) and ICD-9-CM 39.52 (other surgical repair including wrapping)), or endovascular coiling (ICD-9-CM codes 39.72 (endovascular repair of an aneurysm) and 39.79 (other endovascular procedures on other vessels).

Secondary outcomes included hospital cost, hospital complications and length of stay, and. Cost of hospitalization was estimated by applying hospital cost-to-charge ratios provided by HCUP to hospital charges for each discharge. To adjust for inflation, expenditures from 2003 through 2007 were inflated to match the 2008 equivalents using the Consumer Price Index for Medical Services provided by the US Bureau of Labor Statistics [8]. All hospital complications were limited to events that occurred during the index hospital stay. For the definition of sepsis we used AHRQ patient safety indicators “Postoperative Sepsis” and “Central Venous Catheter-Related Blood Stream Infection” criteria [9]. Organ failure associated with sepsis was identified by adding ICD-9-CM codes for acute organ dysfunction to the sepsis diagnosis [10,11]. To identify patients who developed hospital-acquired pneumonia, cardiac arrhythmias and venous thromboembolism we used AHRQ’s Clinical Classification Software (CCS) codes 122, 106 and 118, respectively. This process was developed by AHRQ for researchers to aid in the analysis of large numbers of ICD-9 codes by truncating them from 12,000 diagnosis codes into clinically similar codes for statistical analyses (http://www.hcup-us.ahrq.gov/toolssoftware/ccs/ccs.jsp). Mechanical ventilation was defined by primary or secondary ICD-9-CM procedure codes for continuous mechanical ventilation (96.7x)[12]. Acute kidney injury (AKI) was defined by the presence of any of the following secondary diagnostic codes: 584.5 (acute renal failure [ARF] with lesion of tubular necrosis), 584.6 (ARF with lesion of renal cortical necrosis), 584.7 (ARF with lesion of renal medullary necrosis), 584.8 (ARF with other specified pathologic lesion in kidney), or 584.9 (ARF unspecified) and the additional presence of any of the following procedure codes (in any position): 39.95 (hemodialysis), V45.1 (renal dialysis status), V56.0 (extracorporeal dialysis), or V56.1 (fitting and adjustment of extracorporeal dialysis catheter) indicated renal replacement therapy-required AKI. This approach has been shown to produce high sensitivity and specificity for identification of severe and RRT-requiring AKI but not for less severe AKI [13,14].

**Hospital Characteristics**

Hospitals were described based on location (urban vs. rural), teaching status, bed size and regional information included in the NIS database. We further characterize hospitals’ volume status regarding the annual number of SAH admissions and surgical procedures for SAH as described in previous studies utilizing administrative databases [15,16]. We defined high-volume SAH hospitals as those with >20 admissions for SAH per year and low-volume hospitals as those with <20 admissions per year for SAH [15] used in previous studies examining effect of hospital SAH volume when both surgical and non-surgical admissions were considered. For sensitivity analysis we used cut-off of >80 admissions for SAH per year described in recent study examining effect of SAH volume when only surgically treated SAH were considered [17]. We used ICD-9-CM procedure codes to identify hospitals offering angioplasty for vasospasm with treatment codes ICD-9-CM 00.62 (percutaneous angioplasty of intracranial vessel(s)) and/or ICD-9-CM 39.50 (angioplasty or atherectomy of other non-coronary vessel(s)) as it was previously described and validated [18].

**Statistical Analysis**

We used SURVEY procedures in SAS (v.9.3, Cary, N.C.) and svy commands in Stata (v 12, StataCorp, College Station, TX) to account for stratification, clustering, and unequal weighting of the NIS survey design. Discharge weights provided in the NIS were used to generate nationally representative estimates for the U.S. population. The Taylor series linearization method was used to estimate standard errors [19]. We followed analytic procedures as recommended by AHRQ for subpopulation analyses [20]. For all statistical analyses, the threshold for significance was 0.05.

Frequencies of categorical variables related to patient and hospital characteristics are reported as a weighted numbers (percentage), while continuous variables are reported as mean (standard error). Comparison among variables was made using Wald chi-square test and analysis of variance for categorical and continuous variables, respectively.

Using weighted multivariable logistic and generalized linear regression models we tested for any associations between insurance status and surgical allocation and outcomes, adjusting for patient and hospital factors as explanatory variables. For all models explanatory variables were chosen from domains of the Andersen behavioral model of healthcare utilization [21]. Predisposing characteristics included gender, age, and race. Enabling resources included median household income in ZIP code of residence (based on each patient's ZIP Code, with quartiles based on 1999 demographics [1], hospital characteristics (urban/rural, teaching/community, size, geographic location, volume of ntSAH patients treated and availability of angioplasty) and time of admission. Evaluated health need was represented by the Charlson comorbidity index and presence of six major comorbid conditions (hypertension, diabetes mellitus, chronic lung disease, congestive heart failure, chronic kidney disease and end-stage renal disease). Multivariable logistic regression model for hospital mortality was additionally adjusted for surgical treatment allocation and hospital complications while regression model for cost also included hospital survival status.

Binary outcomes were modeled with multivariable logistic regression models and for each significant covariate we reported odds ratios (OR) with 95% confidence intervals (95% CI). Hospital cost and length of stay were modeled using generalized linear models with gamma and negative binomial-distributed errors, respectively and a log-link function to account for any skewness. Adjusted means for hospital cost and length of stay were reported with 95% CIs for each insurance type. Estimated regression coefficients were exponentiated to represent the cost ratio for each category with respect to the determined reference category for categorical variables or for one unit change in continuous variables. Bonferroni adjustments were used for multiple comparisons. We used the area under the receiver operating characteristics curve (AUC) values and Hosmer-Lemeshow goodness of fit tests to assess model discrimination and fit. Using bootstrapping as a resampling tool we derived 95% CI of AUCs for all logistic regression models. The values corresponding to the 2.5 and 97.5 percentiles of the 1000 bootstrap estimates were used as the limits of the 95% CIs. Statistical significance was defined by a p-value <0.05.

We performed sensitivity analyses by comparing the effect of a) exclusion of transferred patients to adjust for multiple admissions [4], b) exclusion of Medicare patients age < 65 with the highest burden of comorbidities [22], c) inclusion of patients with missing values for race as a separate category, d) exclusion of patients with ESRD, e) exclusion of patients with SAH as a secondary diagnosis, f) exclusion of patients aged 65 years and older as they are almost exclusively insured covered by Medicare and f) omission of different covariates on model fit for each outcome. A difference was considered important if the 95% CIs did not overlap.

**References:**

1. Agency for Healthcare Research and Quality (2008) HCUP Nationwide Inpatient Sample (NIS) Hospital Ownership Files. Healthcare Cost and Utilization Project (HCUP) 2003 - 2008 Rockville, MD.: Agency for Healthcare Research and Quality.

2. Hanmer J, Lu X, Rosenthal GE, Cram P (2014) Insurance Status and the Transfer of Hospitalized PatientsAn Observational Study. Annals of Internal Medicine 160: 81-90.

3. Cram P, Pham HH, Bayman L, Vaughan-Sarrazin MS (2008) Insurance status of patients admitted to specialty cardiac and competing general hospitals: are accusations of cherry picking justified? Med Care 46: 467-475.

4. Shea AM, Reed SD, Curtis LH, Alexander MJ, Villani JJ, et al. (2007) Characteristics of nontraumatic subarachnoid hemorrhage in the United States in 2003. Neurosurgery 61: 1131-1137; discussion 1137-1138.

5. Elixhauser A, Steiner C, Harris DR, Coffey RM (1998) Comorbidity Measures for Use with Administrative Data. Medical Care 36: 8-27.

6. Wald R, Waikar SS, Liangos O, Pereira BJ, Chertow GM, et al. (2006) Acute renal failure after endovascular vs open repair of abdominal aortic aneurysm. J Vasc Surg 43: 460-466; discussion 466.

7. Charlson ME, Pompei P, Ales KL, MacKenzie CR (1987) A new method of classifying prognostic comorbidity in longitudinal studies: development and validation. J Chronic Dis 40: 373-383.

8. United States Bureau of Labor Statistics (2013) Consumer Price Index calculator. Washington, DC United States Department of Labor.

9. Agency for Healthcare Research and Quality (2010) Patient safety indicators: technical specifications. Ver. 4.2. Agency for Healthcare Research and Quality.

10. Dombrovskiy VY, Martin AA, Sunderram J, Paz HL (2005) Facing the challenge: decreasing case fatality rates in severe sepsis despite increasing hospitalizations. Crit Care Med 33: 2555-2562.

11. Vogel TR, Dombrovskiy VY, Carson JL, Graham AM, Lowry SF (2010) Postoperative sepsis in the United States. Ann Surg 252: 1065-1071.

12. Wunsch H, Linde-Zwirble WT, Angus DC, Hartman ME, Milbrandt EB, et al. (2010) The epidemiology of mechanical ventilation use in the United States. Crit Care Med 38: 1947-1953.

13. Waikar SS, Wald R, Chertow GM, Curhan GC, Winkelmayer WC, et al. (2006) Validity of International Classification of Diseases, Ninth Revision, Clinical Modification Codes for Acute Renal Failure. J Am Soc Nephrol 17: 1688-1694.

14. Bihorac A, Yavas S, Subbiah S, Hobson CE, Schold JD, et al. (2009) Long-term risk of mortality and acute kidney injury during hospitalization after major surgery. Ann Surg 249: 851-858.

15. Bardach NS, Zhao S, Gress DR, Lawton MT, Johnston SC (2002) Association Between Subarachnoid Hemorrhage Outcomes and Number of Cases Treated at California Hospitals. Stroke 33: 1851-1856.

16. Bardach NS, Olson SJ, Elkins JS, Smith WS, Lawton MT, et al. (2004) Regionalization of treatment for subarachnoid hemorrhage: a cost-utility analysis. Circulation 109: 2207-2212.

17. Nuno M, Patil CG, Lyden P, Drazin D (2012) The effect of transfer and hospital volume in subarachnoid hemorrhage patients. Neurocrit Care 17: 312-323.

18. Khatri R, Tariq N, Vazquez G, Suri M, Ezzeddine M, et al. (2011) Outcomes After Nontraumatic Subarachnoid Hemorrhage at Hospitals Offering Angioplasty for Cerebral Vasospasm: A National Level Analysis in the United States. Neurocritical Care 15: 34-41.

19. Lee ES, Forthofer RN (2006) Analyzing complex survey data. Thousand Oaks, Calif.: Sage Publications. vii, 91 p. p.

20. HCUP method series report 2003-02. Agency for Healthcare Research and Quality: Agency for Healthcare Research and Quality.

21. Andersen R, Newman JF (2005) Societal and Individual Determinants of Medical Care Utilization in the United States. Milbank Quarterly 83: Online-only-Online-only.

22. Momin EN, Adams H, Shinohara RT, Frangakis C, Brem H, et al. (2012) Postoperative mortality after surgery for brain tumors by patient insurance status in the United States. Arch Surg 147: 1017-1024.
